# Preliminary Tests on the Effects of Atrazine Exposure on the Food-Seeking Behaviors and Locomotion of Juvenile Virile Crayfish (*Faxonius virilis*)

**DOI:** 10.3390/toxics14020164

**Published:** 2026-02-11

**Authors:** Neal D. Mundahl, Darcy E. M. Keyport

**Affiliations:** Ecology and Environmental Science Program, Department of Biology, Winona State University, Winona, MN 55987, USA

**Keywords:** atrazine, crayfish, locomotion, behavior, food-seeking, *Faxonius virilis*

## Abstract

The objective of this study was to conduct preliminary tests to determine if differing concentrations of atrazine affected locomotion and/or food-seeking behaviors of juvenile (second and third instar) virile crayfish after a 4-day (96 h) exposure period. After exposing crayfish to 0, 5, 10, 20, and 100 parts per billion (ppb) atrazine treatments, crayfish were tested and video-taped individually in a flow-through test arena before and during introduction of a food odor. Walking speeds (pre-odor, post-odor, and pre- to post-ratios), time to locate the food-odor source, and success rates in finding the food odor were compared among atrazine treatments. Pre-odor walking speeds, time to locate the food-odor source, and post-odor walking speeds did not differ among the control and treatment crayfish. Crayfish success rates in locating the food-odor source also did not differ among treatments and controls. Crayfish in controls and all atrazine treatments walked slightly, but not significantly, faster after a food odor was presented than before. Virile crayfish food-seeking behavior and locomotion were not affected after exposures up to 100 ppb atrazine, so these behaviors likely are not useful indicators of crayfish exposure to environmentally relevant (5 ppb or less) atrazine levels like those measured periodically in regional streams. Expanded replication and testing may be helpful in assessing the effects of atrazine (especially concentrations at or above 100 ppb) on the food-seeking behaviors of this species, although simple behavioral studies of crayfish may not be sensitive enough to assess the true effects of atrazine on aquatic organisms and communities.

## 1. Introduction

Atrazine is a contact herbicide used to control grassy and broadleaf weeds on croplands since the 1950s [1]. It ranks as one of the most widely used pesticides in the world [2,3], but it has a mixed history due to its persistence in the environment [1,4], its extreme mobility [5], its contamination of both ground and surface water [4,5], and its reported negative effects on wildlife and human health [4,5,6,7,8]. It has been banned by the European Union for nearly 20 years but remains in active use in most other regions around the globe [1,8].

Within the USA, atrazine is used primarily on corn (*Zea mays*) croplands, with the heaviest use occurring within the upper midwestern states comprising the “corn belt” [9,10]. Consequently, this region has been a primary focal area when assessing the environmental fate of atrazine in soils, surface waters, groundwaters, and biota [5,9,10]. There is some evidence that atrazine concentrations were declining in streams during a 10-year period in the early 2000s concomitant with a reduction in the production of corn during that period [10], but atrazine use has again rebounded recently in some areas along with increasing corn hectarage [11], resulting in increased detection frequencies in stream-water samples [12].

There is ongoing disagreement within the scientific community regarding the potential impact of atrazine on the health of aquatic organisms. Numerous studies have reported negative effects of atrazine at environmentally or ecologically relevant concentrations (e.g., <100 ppb) on a variety of aquatic species [5,6,7,8,13,14,15,16]. In contrast, others have reported little to no effect of atrazine on aquatic organisms in the natural environment [9,17,18,19,20]. An ecological risk assessment conducted for atrazine by the United States Environmental Protection Agency concluded that aquatic plant communities are impacted in areas of heaviest atrazine use, and there exists potential chronic risk to amphibians, fish, and aquatic invertebrates in those same locations [4].

A variety of species have been utilized to assess the potential impact of atrazine on aquatic communities. Test organisms have included vascular and non-vascular plants, zooplankton, gastropods, crustaceans, aquatic insects, fish, and amphibians [5,7]. Attempts have also been made to assess the potential impacts of atrazine at the aquatic ecosystem level [9,21]. Overall, these studies suggest that the risks from atrazine can vary widely among organisms and among watersheds, making it difficult to support uniform limits on atrazine applications across wide geographical areas with differing soils and climates [5,9].

With their relatively large size and widespread presence in aquatic habitats around the world [22], crayfish (Malacostraca: Decapoda) have been recommended as excellent bio-indicators because they play key roles in aquatic food-web dynamics [22,23], and they exhibit numerous simple behaviors that may be impacted by pollutants [24,25,26]. Previous studies have reported that various crayfish behaviors, as well as growth rates and physiological processes, were sensitive to numerous pharmaceuticals [27,28,29,30,31], microplastics [32], heavy-metal contamination [33,34], nicotinoid pesticides [35], the herbicide metolachlor [36,37], and ammonia [38]. In addition, the behaviors, growth rates, and biochemical processes of four species of crayfishes have been examined after exposure to atrazine [8,13,14,15,16]. While some behaviors and processes (e.g., molting, locomotion variables, acetylcholinesterase activity) were not affected by atrazine [13,15,16], others (chemosensory behaviors, elevated lactate and alkaline phosphatase in hemolymph) were impacted significantly, with some effects persisting for up to 14 days post-exposure [8,14].

The stream-dwelling virile crayfish, *Faxonius virilis* (Hagen, 1870), has one of the widest geographical distributions of any crayfish species in North America, with native or introduced populations occurring in most US states [39]. Although the species is not used as frequently in biomonitoring experiments as some other native North American species (e.g., rusty crayfish, *Faxonius rusticus*; red swamp crayfish, *Procambarus clarkii*), virile crayfish have been used previously to assess heavy-metal pollution [33] and atrazine impacts [14,16]. However, these atrazine studies either exposed crayfish to only a single atrazine concentration or exposed test subjects to atrazine for <24 h.

Our preliminary study was undertaken to determine if differing concentrations of atrazine affected locomotion (i.e., velocity, both actual and size-relative) and/or food-seeking behaviors of juvenile virile crayfish after a 4-day exposure period. We hypothesized that crayfish locomotion (i.e., velocity) would not be affected by atrazine exposure over the range of concentrations tested, but that exposure to increasing concentrations of atrazine would inhibit food-seeking behavior, requiring crayfish to take more time to locate a food source.

## 2. Methods and Materials

### 2.1. Collection and Acclimation of Virile Crayfish

Free-living juvenile virile crayfish were collected with dip nets from overhanging and submerged shoreline vegetation in Burns Valley Creek, Winona County, MN, USA (44.028537° N, 91.615366° W), during early October. At this time, juvenile crayfish were approximately 4 to 5 months old and likely in their second or third instar based on typical growth patterns for the species [40]. We used juvenile crayfish due to their local abundance and their presumed sensitivity to stressors relative to adult crayfish [15]. Crayfish were placed together in a single laboratory tank containing 100 L of aerated and filtered well water (pH: 7.91–8.08; total hardness: 308–359 mg/L as CaCO_3_; conductivity: 470–533 µS), with three-hole, kiln-fired masonry building bricks (9.2 cm × 5.7 cm × 19.4 cm) provided for shelter. The water temperature was maintained at 15 °C, and lighting was 12 h light and 12 h dark. Crayfish were fed Kaytee Aquatic Turtle Food ad libitum, with uneaten food removed and replaced with fresh food each morning. These conditions were maintained throughout the one-month acclimation period.

### 2.2. Crayfish Exposure to Atrazine

After the one-month acclimation period, crayfish were exposed to four different concentrations of atrazine and two different controls during a single experiment. Atrazine was dissolved in a 3:1 solution of well water and methyl alcohol, and this solution was used to produce concentrations of 5, 10, 20, and 100 parts per billion (ppb) atrazine. These sublethal concentrations were chosen to span most of the range of concentrations used in previous studies of atrazine effects on this and other species of crayfish [8,13,14,15,16]. Exposure containers held 2 L of each test solution. The two controls consisted of (1) all well water, and (2) a well water and methyl alcohol solution containing the same alcohol concentration (1.85 mL methyl alcohol/L) as the atrazine exposure containers. Test containers held six to nine crayfish each (crayfish were randomly assigned to the different treatments, although some attempt was made to balance sex ratios within each treatment), and were aerated continuously for the 4-day (or 96 h) exposure period. A 96 h exposure period was used to align this study with those that used acute [8,36] rather than chronic (e.g., 10 to 14 days) [27,30,31,35] exposure to study crayfish responses to environmental contaminants. In addition, the 96 h exposure period matched the length of time over which Minnesota agencies collect composite stream water samples for contaminant analysis [12]. Crayfish were not fed during the exposure period to enhance the food-seeking response [13,14,36] and were provided with bricks as shelter. Treatment atrazine concentrations were not measured analytically during the exposure period. Although atrazine has a potential for sorption, degradation, and dilution in aquatic environments over time [41], concentrations in clean crayfish-exposure aquaria can remain relatively stable (i.e., ±5% of intended concentrations) for up to two weeks [8].

### 2.3. Testing Container and Food Odor

The testing container consisted of a transparent rectangular plastic tub (15 cm wide × 23 cm long × 11 cm deep), with an outflow tube at one end for maintaining testing volume at 2 L and an inflow tube at the opposite end positioned at approximately the 1 L level. An aerated well-water reservoir connected to the inflow tube was adjusted to maintain a flow rate of 125 mL/min into the testing chamber. The bottom of the testing container was gridded (1 cm grids) to aid in movement analyses, and the removeable top was a sheet of clear plastic. A small, removeable, cylindrical, wire-mesh cage was used to confine a crayfish until testing began. A video camera was positioned directly above the testing container to record each trial.

A food-odor stimulus for use in testing was prepared by homogenizing 5 g of frozen sockeye salmon (*Oncorynchus nerka*) in 1 L of well water. This food-odor stimulus was refrigerated until use in each trial. Crayfish not included in the experiment readily consumed salmon after feeding only on turtle food for one month.

### 2.4. Testing Crayfish Food-Locating Behavior

To test each crayfish, 2 L of aerated well water was added into the testing container and a single crayfish was selected randomly from among the various treatments and controls and placed in the testing container and restrained within the mesh cage. Water inflow was initiated and continued for 2 min while the crayfish was restrained. The crayfish was then released, videotaping was begun, and the crayfish’s movements throughout the chamber were recorded for 2 min. After returning the crayfish back to the starting position and placing it in the cage, the food-odor stimulus was injected at a rate of 1 mL/10 s into the inlet tube using a 20 mL syringe. Crayfish movements again were videotaped for 2 min or until the crayfish successfully located the food-source inlet (or approached it to within a 5 cm radius). The 2 min videotaping period was established after preliminary tests indicated that most crayfish located the food source in <2 min. After testing, the crayfish was removed, its sex determined, and its carapace length was measured (nearest 0.1 mm) with dial calipers. This process was repeated until all crayfish had been tested.

### 2.5. Videotape and Data Analyses

Video tapes from each test were examined manually (no video analysis software was used; movements assessed via on-screen timer and gridded background) to assess crayfish food-seeking behavior after atrazine exposure. Manual analyses of videotaped behaviors may introduce bias due to human error, subjectivity, and observational boredom leading to missed behaviors [42]. However, manual analyses can be suitable when observations are made on only a single subject at a time, when observation times are relatively short, and when behaviors being monitored occur in only one or two dimensions [42,43]. Measuring linear distances traveled by single crayfish for a 2 min time period fit these criteria.

Videotape analyses were used to determine the proportion of crayfish which successfully located the food source, the length of time it took them to reach the food sources, time spent stationary or motionless (determined as number of 10-s time periods with no movement), and the walking speeds before and after release of the food odor. Walking speeds were examined both as an actual velocity (cm traveled/10 s) and as a standardized velocity based on crayfish size (carapace lengths/10 s). Success-rate data were analyzed with a Chi-square goodness of fit test. Walking speeds, time spent motionless, and time to find the food-odor source were analyzed with non-parametric Kruskal–Wallis tests to determine if any differences in crayfish food-locating behavior existed among the various control and atrazine exposure concentrations. Walking speeds also were compared before and after release of the food odor using separate Wilcoxon signed-ranks tests for each treatment.

## 3. Results

### 3.1. Crayfish Size and Sex

Of the 39 crayfish used in experiments, 22 were female and 17 were male. Despite slightly more females than males tested, sex ratios did not differ significantly from 1:1 across the various treatments (Chi-square = 2.85, df = 5, *p* = 0.723). In addition, the size of crayfish (mean carapace lengths) did not differ significantly among treatments (Kruskal–Wallis *H* = 4.07, *p* = 0.40), with a median size of 14.7 mm (range = 10.4 to 20.9 mm) overall.

### 3.2. Pre-Odor Activity

Before exposure to food odor, individual crayfish exhibited actual walking speeds ranging from 0.9 to 10.4 cm/10 s. Crayfish exposed to differing concentrations of atrazine did not display significant differences (*p* = 0.17) in simple walking speeds (Table 1).

When walking speeds were standardized on a basis of body size, crayfish still exhibited a wide range of speeds, from 0.5 to 5.8 carapace lengths/10 s. Crayfish exposed to most treatments and controls moved at rates of two to four carapace lengths/10 s (Table 1). As with simple walking speeds, there was no significant difference (*p* = 0.13) in standardized walking speeds among treatments (Table 1).

Individual crayfish often remained motionless during the 2 min time period prior to odor introduction. Although many crayfish were active during every 10 s time period, some individuals spent up to six time periods (60 s, 50% of the pre-odor observation period) motionless. Crayfish exposed to differing concentrations of atrazine displayed no significant difference in time periods spent motionless (Table 1).

### 3.3. Odor Detection

**Figure 1:**
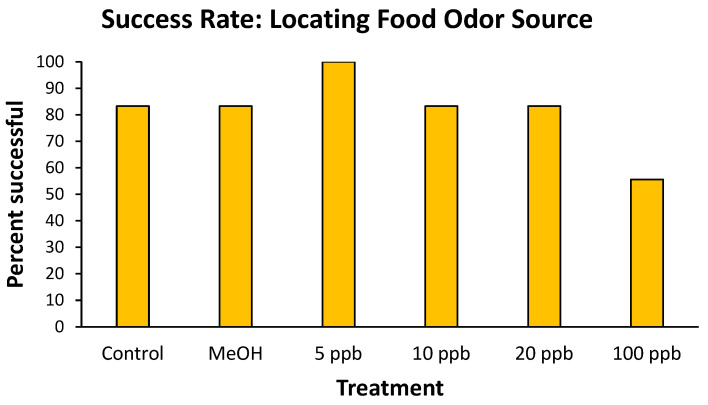
Success rates of virile crayfish in locating a food-odor source after 4-day exposures to differing atrazine treatments. MeOH was a methanol control.

Overall, 80% of the crayfish tested were successful in locating the food-odor source during the 2 min observation period, locating the source in a median of 42 s. There was no significant difference in the time for successful crayfish to locate the food source among the test treatments (Table 1). Similarly, there was no significant difference (Chi-square = 4.93, df = 5, *p* = 0.43) in the success rate of crayfish among treatments (Figure 1).

### 3.4. Post-Odor Activity

In the presence of food odor, individual crayfish exhibited actual walking speeds ranging from 2.2 to 20.2 cm/10 s. There was no significant difference in the walking speeds of crayfish exposed to different treatments (Table 1).

Standardized walking speeds in the presence of food odor ranged from 1.1 to 14.3 carapace lengths/10 s, with most crayfish in the various treatments moving at a median rates of 3 to 7 carapace lengths/10 s (Table 1). Again, there was no significant difference in standardized walking speeds among the atrazine treatments and controls (Table 1).

**Figure 2:**
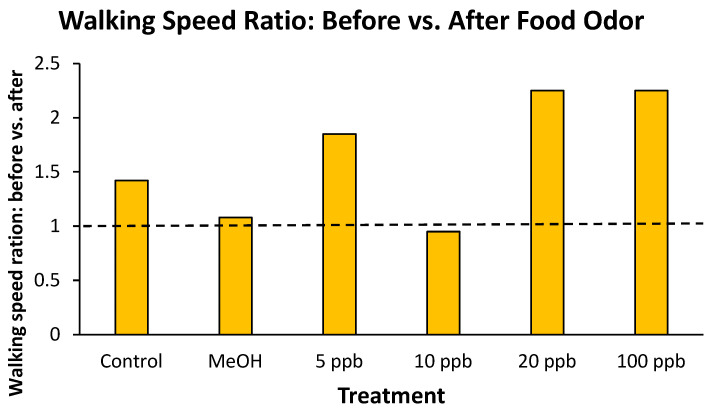
Ratios of virile crayfish walking speeds before and after being presented with a food-odor source. Values are medians. The horizontal dashed line represents a 1:1 ratio, or no change in walking speed.

When individual crayfish walking speeds in the presence of food odor were compared to walking speeds of those crayfish in the absence of odor, they all appeared to have increased their speed (Figure 2). However, when before versus after walking speeds were compared treatment by treatment, walking speeds did not differ in the presence or absence of a food odor (all Wilcoxon signed ranks tests *p* > 0.25). Across all controls and treatments, no significant difference in before versus after walking speed ratios (Kruskal–Wallis *H* = 6.03, *p* = 0.20) was detected among treatments and controls.

## 4. Discussion

This preliminary study produced three findings related to atrazine exposure and the food-seeking behavior of juvenile virile crayfish. First, the success of juvenile crayfish in locating a food-odor source was not diminished in crayfish exposed to atrazine concentrations up to 100 ppb for 96 h. Second, juvenile crayfish walking speeds were not altered in crayfish exposed to any of the atrazine concentrations tested. Finally, juvenile crayfish exposed to atrazine did not require more time to locate a food-odor source compared to control crayfish with no atrazine exposure.

The chemosensory ability of crayfish to detect a food source is a necessity for their basic survival. Chemoreceptors are located on the antennae and various portions of the body surface, where they detect chemicals released into the water from potential foods [25,43]. Anthropogenic chemicals and compounds can interfere with these chemoreceptors, reducing their sensitivity to the natural stimuli [25,44]. Previous studies have reported that food-seeking behavior of crayfish was compromised after exposure to various pollutants, such as acids [45], copper [46], and different agricultural herbicides [13,14,36,37]. In the present study, juvenile crayfish exposed to atrazine did not display reduced success in locating the food-odor source. This result disagrees with two previous studies which reported that crayfish exposed to 80 ppb atrazine were less successful at locating a food source than were control crayfish [13,14]. The reason for this difference is unknown, although our study differed from previous atrazine studies which exposed only larger crayfish to atrazine, used only a single atrazine concentration, and/or exposed test subjects to atrazine for <24 h [13,14,16]. At best, juvenile crayfish in our study exposed to 100 ppb atrazine exhibited only a slight (but not significant) tendency toward reduced success in locating a food-odor source. Regardless, atrazine concentrations of 80 ppb or higher may be present in stream water in some regions where atrazine use is heavy [9], but the State of Minnesota did not report a stream-water atrazine concentration (based on 96 h composite samples) exceeding 26 ppb from among 9043 samples analyzed during the period 2007 through 2023 [12]. During that time, only 15 samples (<0.2% of samples examined) had atrazine concentrations exceeding the state chronic standard of 10 ppb. Consequently, atrazine concentrations at levels reported in other studies [13,14,16] to inhibit food location by crayfish appear to be extremely rare to non-existent in our region of the upper midwestern USA.

Crayfish can display altered locomotor activity after exposure to various contaminants [24]. Increased locomotion, activity, boldness, and/or aggression has been reported in crayfish exposed to zinc [34] and two different antidepressant pharmaceuticals [30,31], whereas decreased activities have been associated with crayfish exposure to the herbicide metolachlor [36], nicotinoid pesticides [35], a third antidepressant [27], and a mixture of six different antidepressants [29]. In our study, the walking speeds of juvenile crayfish were not significantly reduced in individuals exposed to atrazine in concentrations up to 100 ppb. This result agrees with other studies of atrazine that found crayfish locomotion was not affected by exposure to 80 ppb atrazine for 72 or 96 h [13,14]. However, a different study reported reduced motor activity in crayfish exposed to 1.21 mg/L (1210 ppb) atrazine for two weeks [8]. Based on these somewhat conflicting results, we suspect that there may be some threshold atrazine exposure concentration between 100 and 1000 ppb, for a period of 96 h or longer, that might produce locomotor impairment in crayfish.

In our study, we hypothesized that prior exposure to atrazine would not affect the walking speeds of juvenile crayfish. This hypothesis was supported for crayfish both prior to and after being presented with a food odor. We anticipated, however, that crayfish might increase their walking speeds after a food odor was presented, as they did in an earlier study [36]. Increased walking speeds in the presence of a food odor should not be surprising, as such a response would indicate recognition of and movement toward potential food [25,44]. However, our study found no significant increase in walking speeds of individual crayfish after a food odor was introduced, regardless of atrazine exposure concentration. Interestingly, increased crayfish walking speeds observed previously after introducing a food-odor source did not result in less time needed by these crayfish to find the food-odor source [36], suggesting that movement was faster, but less directed, indicating heightened but less productive activity when a food source was sensed.

Juvenile crayfish used in our study may differ from adult crayfish in their response to atrazine exposure, preventing us from extrapolating our lack of atrazine effects on food-seeking behaviors of juveniles to other age classes of virile crayfish. Juvenile crayfish may display lower tolerances than adults when exposed to various physical and chemical stressors such as elevated water temperatures [47], low pH [48,49], increased salinities [50,51], and elevated concentrations of heavy metals [52]. However, adult crayfish may be just as sensitive as juveniles to some stressors [53], especially during periods of molting [52]. Until further studies of the effects of atrazine on food-seeking behavior of both juvenile and adult virile crayfish can be conducted, it should not be assumed that adult crayfish will respond similarly to juveniles when exposed to atrazine.

Overall, our preliminary study agreed with previous research [8,13,15,16] suggesting that environmentally realistic atrazine treatment concentrations (e.g., <20 ppm) like those found periodically in streams within our region would have little to no observable effect on the specific crayfish behaviors examined. However, this does not imply that atrazine concentrations < 20 ppm in stream water have no effect(s) on aquatic life. As has been reported by others, even atrazine concentrations < 1 ppb can have negative effects on physiological processes and reproduction in aquatic organisms [1,4,7,54,55,56,57,58], including at the tissue-, cellular-, and sub-cellular levels in crayfish [59,60,61,62]. Consequently, continued monitoring, management, and regulation of atrazine applications to agricultural crops, and continued study of atrazine’s potential effects on aquatic organisms, are warranted to adequately safeguard aquatic communities [1,4]. Additionally, ongoing efforts to decrease the impact of atrazine in aquatic systems [63] and develop treatment technologies to remove it from the environment [64] should be encouraged.

We consider our study to be preliminary in nature, due to (1) the lack of repeated experimental trials and (2) the focus only on juvenile crayfish. Our desire to evaluate the effects of a wide range of atrazine concentrations on crayfish food-seeking behavior while maintaining statistically adequate sample sizes at each concentration precluded us from conducting multiple trials, as seasonal environmental changes (rapidly decreasing stream-water temperatures) prevented us from collecting additional animals for further testing. Given the sensitivity and short-term duration of behavioral toxicity testing relative to many other toxicity testing approaches (e.g., acute-lethality, developmental, or reproductive endpoints), having sufficient trial replication to maximize statistical power is strongly recommended [65]. We also were unable to collect sufficient numbers of adult crayfish from our study stream to fill out a test regime involving both controls and varying atrazine concentrations. Although other studies have assessed only juvenile or only adult crayfish after herbicide exposures [13,59,60,61,62], we feel that a single comprehensive study encompassing multiple crayfish age groups exposed to the same testing conditions would best address the relative tolerances of different-age crayfish to atrazine exposure. We expect that our future experiments assessing atrazine exposure effects on virile crayfish will include both juvenile and adult organisms sufficient in number to produce statistically strong comparisons between the age groups.

## 5. Conclusions

This preliminary assessment of atrazine effects on juvenile virile crayfish indicated that environmentally relevant atrazine concentrations had no observable effects on either crayfish food-seeking behaviors or locomotion. Juvenile virile crayfish locomotion and success in locating food sources was not affected after exposures to atrazine concentrations up to 100 ppb. Replicated experiments including both juvenile and adult virile crayfish exposed to a wider range of atrazine concentrations will be needed to more completely investigate the effects of atrazine on locomotion and food-seeking behaviors of this species.

## Figures and Tables

**Table 1:** Measured variables of virile crayfish after 4-day exposures to differing treatments of atrazine (5, 10, 20, 100 ppb) and controls. Values are medians with minima and maxima in parentheses. Results of Kruskal–Wallis comparisons among treatments for each variable before and after presentation of food odor are shown. MeOH was a methanol control. Actual walking speed in cm/10 s, standardized walking speed in carapace lengths/10 s. Numbers of time periods spent motionless are indicated out of a possible twelve, 10 s periods per crayfish. Time to locate food-odor source is shown in seconds.

Variable	Control	MeOH	5 ppb	10 ppb	20 ppb	100 ppb	K-W *H*	*p* Value
*Before food odor*								
Actual walking speed	3.8 (0.9, 10.3)	4.1 (3.0, 6.1)	4.6 (1.0, 6.6)	5.5 (4.6, 7.2)	5.8 (0.9, 10.4)	3.1 (0.1, 8.6)	7.92	0.17
Standardized walking speed	2.6 (0.5, 4.9)	3.3 (0.6, 4.1)	2.7 (0.7, 5.2)	4.0 (3.4, 5.3)	3.9 (0.6, 5.8)	2.9 (0.6, 5.7)	8.05	0.13
10 s periods motionless	3 (1, 5)	3 (1, 6)	4 (1, 5)	3 (1, 6)	2 (1, 3)	3 (0, 4)	4.35	0.51
*After food odor*								
Actual walking speed	5.9 (4.6, 6.7)	5.8 (2.7, 15.3)	6.0 (2.4, 19.8)	8.6 (3.3, 10.7)	8.8 (2.2, 20.2)	4.6 (2.6, 9.1)	4.75	0.31
Standardized walking speed	3.6 (1.7, 9.7)	3.9 (2.2, 5.2)	4.5 (1.1, 14.1)	4.7 (1.5, 9.3)	6.1 (1.5, 14.3)	2.7 (1.2, 4.5)	3.95	0.61
Time to locate food-odor source	39 (17, 50)	50 (31, 88)	47 (24, 94)	37 (5, 48)	29 (13, 84)	39 (11, 48)	1.84	0.77

## Data Availability

The original contributions presented in this study are included in the article. Further inquiries can be directed to the corresponding author.

## References

[1] Jablonowski N.D., Schäffer A., Burauel P. (2011). Still present after all these years: Persistence plus potential toxicity raise questions about the use of atrazine. Environ. Sci. Pollut. Res..

[2] Cunningham W.P., Cunningham M.A., Saigo B.W. (2007). Environmental Science: A Global Concern.

[3] Withgott J., Brennan S. (2006). Environment: The Science Behind the Stories.

[4] Farruggia F.T., Rossmeisi C.M., Hetrick J.A., Biscoe M. (2016). Refined Ecological Risk Assessment for Atrazine.

[5] Graymore M., Stagnitti F., Allinson G. (2001). Impacts of atrazine in aquatic ecosystems. Environ. Int..

[6] Hayes T.B. (2004). There is no denying this: Defusing the confusion about atrazine. BioScience.

[7] Hayes T.B., Khoury V., Narayan A., Nazir M., Park A., Brown T., Adame L., Chan E., Bucholz D., Stueve T. (2010). Atrazine induces complete feminization and chemical castration in male African clawed frogs (*Xenopus laevis*). Proc. Natl. Acad. Sci. USA.

[8] Stara A., Kouba A., Velisek J. (2018). Biochemical and histological effects of sub-chronic exposure to atrazine in crayfish *Cherax destructor*. Chem. Biol. Interact..

[9] Solomon K.R., Baker D.B., Richards P., Dixon K.R., Klaine S.J., La Point T.W., Kendall R.J., Weisskopf C.P., Giddings J.M., Giesy J.P. (1996). Ecological risk assessment of atrazine in North American surface waters. Environ. Toxicol. Chem..

[10] Ryberg K.R., Stone W.W., Baker N.T. (2020). Causal factors for pesticide trends in streams of the United States: Atrazine and deethylatrazine. J. Environ. Qual..

[11] Annan K., Fausti S.W., Van der Sluis E., Kolady D.E. (2024). Corn acreage intensification levels in U.S. corn belt states. J. Agric. Appl. Econ..

[12] Minnesota Department of Agriculture (2024). 2023 Water Quality Monitoring Report.

[13] Belanger R.M., Peters T.J., Sabhapathy G.S., Khan S., Katta J., Abraham N.K. (2015). Atrazine exposure affects the ability of crayfish (*Orconectes rusticus*) to localize a food odor source. Arch. Environ. Contam. Toxicol..

[14] Belanger R.M., Mooney L.N., Nguyen H.M., Abraham N.K., Peters T.J., Kana M.A., May L.A. (2015). Acute atrazine exposure has lasting effects on chemosensory responses to food odors in crayfish (*Orconectes virilis*). Arch. Environ. Contam. Toxicol..

[15] Mac Loughlin C., Canosa I.S., Silveyra G.R., López Greco L.S., Rodriguez E.M. (2016). Effects of atrazine on growth and sex differentiation, in juveniles of the freshwater crayfish *Cherax quadricarinatus*. Ecotoxicol. Environ. Saf..

[16] Steele A.N., Belanger R.M., Moore P.A. (2018). Exposure through runoff and groundwater contamination differentially impact behavior and physiology of crustaceans in fluvial systems. Arch. Environ. Contam. Toxicol..

[17] Solomon K.R., Carr J.A., Du Preez L.H., Giesy J.P., Kendall R.J., Smith E.E., Van Der Kraak G.J. (2008). Effects of atrazine on fish, amphibians, and aquatic reptiles: A critical review. Crit. Rev. Toxicol..

[18] Van Der Kraak G.J., Hosmer A.J., Hanson M.L., Kloas W., Solomon K.R. (2014). Effects of atrazine in fish, amphibians, and reptiles: An analysis based on quantitative weight of evidence. Crit. Rev. Toxicol..

[19] Hanson M.L., Solomon K.R., Van Der Kraak G.J., Brian R.A. (2019). Effects of atrazine on fish, amphibians, and reptiles: Update of the analysis based on quantitative weight of evidence. Crit. Rev. Toxicol..

[20] Smith P.N., Armbrust K.L., Brain R.A., Chen W., Galic N., Ghebremichael L., Giddings J.M., Hanson M.L., Maul J., Van Der Kraak G. (2021). Assessment of risks to listed species from the use of atrazine in the USA: A perspective. J. Toxicol. Environ. Health B.

[21] Detenbeck N.E., Hermanutz R., Allen K., Swift M.C. (1996). Fate and effects of the herbicide atrazine in flow-through wetland mesocosms. Environ. Toxicol. Chem..

[22] Kawai T., Faulkes Z., Scholtz G. (2015). Freshwater Crayfish: A Global Overview.

[23] Hill A.M., Lodge D.M. (1999). Replacement of resident crayfishes by an exotic crayfish: The roles of competition and predation. Ecol. Soc. Am..

[24] Hebel D.K., Jones M.B., Depledge M.H. (1997). Responses of crustaceans to contaminant exposure: A holistic approach. Estuar. Coast. Shelf Sci..

[25] Olsén K.H., Breithaupt T., Thiel M. (2011). Effects of pollutants on olfactory mediated behaviors in fish and crustaceans. Chemical Communication in Crustaceans.

[26] Kuklina I., Kouba A., Kozák P. (2013). Real-time monitoring of water quality using fish and crayfish as bio-indicators: A review. Environ. Monit. Assess..

[27] Tierney A.J., Hanzlik K.N., Hathaway R.M., Powers C., Roy M. (2016). Effects of fluoxetine on growth and behavior in the crayfish *Orconectes rusticus*. Mar. Freshw. Behav. Physiol..

[28] Guo W., Hossain M.S., Kubec J., Grabicová K., Randák T., Burič M., Kouba A. (2020). Psychoactive compounds at environmental concentration alter burrowing behavior in the freshwater crayfish. Sci. Total Environ..

[29] Hossain M.S., Kubec J., Guo W., Roje S., Ložek F., Grabicová K., Randák T., Kouba A., Burič M. (2021). A combination of six psychoactive pharmaceuticals at environmental concentrations alters the locomotory behavior of clonal marbled crayfish. Sci. Total Environ..

[30] Reisinger A.J., Reisinger L.S., Richmond E.K., Rosi E.J. (2021). Exposure to a common antidepressant alters crayfish behavior and has potential subsequent ecosystem impacts. Ecosphere.

[31] Suryanto M.E., Luong C.T., Vasquez R.D., Roldan M.J.M., Hung C.-H., Ger T.-R., Hsiao C.-D. (2023). Using crayfish behavior assay as a simple and sensitive model to evaluate potential adverse effects of water pollution: Emphasis on antidepressants. Ecotoxicol. Environ. Safety.

[32] Pastorino P., Anselmi S., Zanoli A., Esposito G., Bondavalli F., Dondo A., Puci A., Pizzul E., Faggio C., Barceló D. (2023). The invasive red swamp crayfish (*Procambarus clarkia*) as a bioindicator of microplastic pollution: Insights from Lake Candia (northwestern Italy). Ecol. Indic..

[33] Brittle S.W., Paluri S.L.A., Foose D.P., Ruis M.T., Amato M.T., Lam N.H., Buttigieg B., Gagnon Z.E., Sizemore I.E. (2016). Freshwater crayfish: A potential benthic-zone indicator of nanosilver and ionic silver pollution. Environ. Sci. Technol..

[34] Mamdouh S., Mohamed A.S., Mohamed H.A., Fahmy W.S. (2022). Zn contamination stimulates agonistic behavior and oxidative stress of crayfishes (*Procambarus clarkii*). J. Trace Elem. Med. Biol..

[35] Sohn L., Brodie R.J., Couldwell G., Demmons E., Sturve J. (2018). Exposure to a nicotinoid pesticide reduces defensive behaviors in a non-target organism, the rusty crayfish *Orconectes rusticus*. Ecotoxicology.

[36] Wolf M.C., Moore P.A. (2002). Effects of the herbicide metolachlor on the perception of chemical stimuli by *Orconectes rusticus*. J. N. Am. Benthol. Soc..

[37] Alacantara F., Weighman K.K., Moore P.A. (2019). Variable background flow on aquatic toxicant exposure alters foraging patterns on crayfish. Bull. Environ. Contam. Toxicol..

[38] Edwards D.D., Klotz K.L., Moore P.A. (2018). Exposure to sublethal ammonia concentrations alters the duration and intensity of agonistic interactions in the crayfish, *Orconectes rusticus*. Bull. Environ. Contam. Toxicol..

[39] Durland Donahou A. (2024). Faxonius virilis (Hagen, 1870).

[40] Simon T.P., Stewart C.R. (2014). Growth, length-weight relationships, and condition associated with gender and sexual stage in the invasive northern crayfish, *Orconectes virilis* Hagen, 1870 (Decapoda, Cambaridae). Proc. Indiana Acad. Sci..

[41] Mersie W., McNamee C., Seybold C.A., Tierney D.P. (2000). Diffusion and degradation of atrazine in a water/sediment system. Environ. Toxicol. Chem..

[42] Anderson D.J., Perona P. (2014). Toward a science of computational ethology. Neuron.

[43] Luxem K., Sun J.J., Bradley S.P., Krishnan K., Yttri E., Zimmerman J., Pereira T.D., Laubach M. (2023). Open-source tools for behavioral video analysis: Setup, methods, and best practices. eLife.

[44] Kubec J., Kouba A., Burič M. (2019). Communication, behaviour, and decision making in crayfish: A review. Zool. Anz..

[45] Tierney A.J., Atema J. (1986). Effects of acidification on the behavioral response of crayfishes (*Orconectes virilis* and *Procambarus acutus*) to chemical stimuli. Aquat. Toxicol..

[46] Sherba M., Dunham D.W., Harvey H.H. (2000). Sublethal copper toxicity and food response in the freshwater crayfish *Cambarus bartonii* (Cambaridae, Decapoda, Crustacea). Ecotoxicol. Environ. Saf..

[47] Cox D.K., Beauchamp J.J. (1982). Thermal resistance of juvenile crayfish, *Cambarus bartoni* (Fabricus): Experiment and model. Am. Midl. Nat..

[48] France R.L. (1984). Comparative tolerance to low pH of three life stages of the crayfish *Orconectes virilis*. Can. J. Zool..

[49] DiStefano R.J., Neves R.J., Helfrich L.A., Lewis M.C. (1991). Response of the crayfish *Cambarus bartonii bartonii* to acid exposure in southern Appalachian streams. Can. J. Zool..

[50] Mills B.J., Geddes M.C. (1980). Salinity tolerance and osmoregulation of the Australian freshwater crayfish *Cherax destructor* Clark (Decapoda: Parastacidae). Australian J. Mar. Freshw. Res..

[51] Holdich D.M., Harlioğlu M.M., Firkins I. (1997). Salinity adaptations of crayfish in British waters with particular reference to *Austropotamobius pallipes*, *Astacus leptodactlyus* and *Pacifasticus leniusculus*. Estuar. Coast. Shelf Sci..

[52] Wigginton A.J., Birge W.J. (2007). Toxicity of cadmium to six species in two genera of crayfish and the effect of cadmium on molting success. Environ. Toxicol. Chem..

[53] Mundahl N.D., Benton M.J. (1990). Aspects of the thermal ecology of the rusty crayfish *Orconectes rusticus* (Girard). Oecologia.

[54] Langlois V.S., Carew A.C., Pauli B.D., Wade M.G., Cooke G.M., Trudeau V.I. (2010). Low levels of the herbicide atrazine alter sex ratios and reduce metamorphic success in *Rana pipiens* tadpoles raised in outdoor mesocosms. Environ. Health Perspect..

[55] Lenkowski J.R., McLaughlin K.A. (2010). Acute atrazine exposure disrupts matrix metalloproteinases and retinoid signaling during organ morphogenesis in *Xenopus laevis*. J. Appl. Toxicol..

[56] Olivier H.M., Moon B.R. (2010). The effects of atrazine on spotted salamander embryos and their symbiotic alga. Ecotoxicology.

[57] Rohr J.R., McCoy K.A. (2010). A qualitative meta-analysis reveals consistent effects of atrazine on freshwater fish and amphibians. Environ. Health Perspect..

[58] Tillitt D.E., Papoulias D.M., Whyte J.J., Richter C.A. (2010). Atrazine reduces reproduction in fathead minnow (*Pimephales promelas*). Aquat. Toxicol..

[59] Abdulelah S.A., Crile K.G., Almouseli A., Awali S., Tutwiler A.Y., Tien E.A., Manzo V.J., Hadeed M.N., Belanger R.M. (2020). Environmentally relevant atrazine exposures cause DNA damage in cells of the lateral antennules of crayfish (*Faxonius virilis*). Chemosphere.

[60] Hadeed M.N., Castiglione C.L., Saleem S., Chammout D.H., Muskovac M.D., Crile K.G., Abdulelah S.A., Mallhagh-Fard A., Rampari E.Y., Grabowski G.M. (2022). Environmentally relevant atrazine exposure leads to increases in DNA damage and changes in morphology in the hepatopancreas of crayfish (*Faxonius virilis*). Environ. Adv..

[61] Coy C.O., Steele A.N., Abdulelah S.A., Belanger R.M., Crile K.G., Stevenson L.M., Moore P.A. (2022). Differing behavioral changes in crayfish and bluegill under short- and long-chain PFAS exposures: Field study in Northern Michigan, USA. Ecotoxicol. Environ. Saf..

[62] Reddy S.G., Muskovac M.D., Alduis A., Manns J.C., Awali S., Hanna A., Jacob L.L., Ibrahim P., Alsharifi H., Vosbigian G. (2025). Combined exposure to ecologically relevant concentrations of atrazine and microcystin causes morphological changes in the hepatopancreas of crayfish. Integrat. Compar. Biol..

[63] de Albuquerque F.P., de Oliveira J.L., Moschini-Carlos V., Fraceto L.F. (2020). An overview of the potential impacts of atrazine in aquatic environments: Perspectives for tailored solutions based on nanotechnology. Sci. Total Environ..

[64] He H., Liu Y., You S., Liu J., Xiao H., Tu Z. (2019). A review on recent treatment technology for herbicide atrazine in contaminated environment. Int. J. Environ. Res. Public Health.

[65] Melvin S.D., Wilson S.P. (2013). The utility of behavioral studies for aquatic toxicology testing: A meta-analysis. Chemosphere.

